# A stable and quantitative method for dimensionality reduction of aortic centerline

**DOI:** 10.3389/fcvm.2022.940711

**Published:** 2022-08-31

**Authors:** Tao Peng, Hongji Pu, Peng Qiu, Han Yang, Ziyue Ju, Hui Ma, Juanlin Zhang, Kexin Chen, Yanqing Zhan, Rui Sheng, Yi Wang, Binshan Zha, Yang Yang, Shu Fang, Xinwu Lu, Jinhua Zhou

**Affiliations:** ^1^School of Biomedical Engineering, Anhui Medical University, Hefei, China; ^2^Department of Vascular Surgery, Shanghai Ninth People’s Hospital Affiliated to Shanghai Jiao Tong University School of Medicine, Shanghai, China; ^3^The Fourth Affiliated Hospital of Anhui Medical University, Hefei, China; ^4^Chaohu Clinical Medical College, Anhui Medical University, Hefei, China; ^5^Department of Vascular and Thyroid Surgery, The First Affiliated Hospital of Anhui Medical University, Hefei, China; ^6^Department of General Surgery, The First Affiliated Hospital of Anhui Medical University, Hefei, China; ^7^Department of Computer Science and Engineering, Shanghai Jiao Tong University, Shanghai, China; ^8^3D-Printing and Tissue Engineering Center, Anhui Provincial Institute of Translational Medicine, Anhui Medical University, Hefei, China

**Keywords:** aortic dissection, centerline modeling, geometric plane projection, morphological analysis, three-dimensional coordinate transformation

## Abstract

Aortic dissection (AD) is a fatal aortic disease with high mortality. Assessing the morphology of the aorta is critical for diagnostic and surgical decisions. Aortic centerline projection methods have been used to evaluate the morphology of the aorta. However, there is a big difference between the current model of primary plane projection (PPP) and the actual shape of individuals, which is not conducive to morphological statistical analysis. Finding a method to compress the three-dimensional information of the aorta into two dimensions is helpful to clinical decision-making. In this paper, the evaluation parameters, including contour length (CL), enclosure area, and the sum of absolute residuals (SAR), were introduced to objectively evaluate the optimal projection plane rather than artificial subjective judgment. Our results showed that the optimal projection plane could be objectively characterized by the three evaluation parameters. As the morphological criterion, SAR is optimal among the three parameters. Compared to the optimal projection plane selected by traditional PPP, our method has better AD discrimination in the analysis of aortic tortuosity, and is conducive to the clinical operation of AD. Thus, it has application prospects for the preprocessing techniques for the geometric morphology analysis of AD.

## Introduction

Aortic dissection (AD) refers to a pathological state in which the blood in the aortic lumens flows into the outer layer or the junction of the aortic media through the tear of the aortic intima. It makes the aorta into two layers, causes a true lumen and a false lumen, and extends along the longitudinal axis of the aorta (1–3). AD will cause the false lumen to compress the true lumen, which results in ischemic changes in the essential organs and severe complications (4–6). With the improvement of the medical level, the diagnosis and treatment of cardiovascular disease have made significant progress, but AD is still a fatal aortic disease (7–9).

The formation of AD is based on biomechanical changes in the geometry of the aorta. In some parts of the aorta, the force of the tear is larger than the force of the cohesion (10). To monitor the formation and development of AD, it is necessary to observe the geometrical change of the aorta. On the one hand, the geometric changes of the aorta are a macroscopic marker of physiological changes in the aorta. On the other hand, the changes in the shape of the aorta can induce abnormal fluid dynamics of blood flow which may aggravate the formation and development of aortic diseases (10–12). Traditional medical images are primarily based on slice computed tomography (CT) scan, which cannot directly provide geometric morphological analysis of the aorta. The three-dimensional (3D) morphology of the aorta has been observed and analyzed, and the corresponding hemodynamic parameters were obtained to provide more information for AD imaging analysis (13, 14). However, the knowledge and modeling formulas involved in these researches are so complex that to have critical requirements for the theoretical basis. The continuous improvements of the models are also high-cost, which limits wide applications in clinical practice.

To reduce the complexity of 3D morphological analysis, some dimensionality reduction methods have been applied to extract the morphological features of the aorta. The geometric coordinates modeling method can make a concise and explicit expression of morphological characteristics of the object without obscure physical formulas and reduce the difficulty of research and expand its applications. A mathematical model was put forward to analyze Type A AD and ascending aortic aneurysms, in which a new geometric parameter, aortic curvature (AC), was defined and had good performance for prediction (15). Based on geometric mathematical modeling, Patterson et al. (16) compared the diameter and area of the reconstructed aorta with those in reality. The results illustrated no significant change in the aortic morphology after centerline extraction and verified the feasibility of aortic reconstruction in the clinical. These attempts provide another potential option for aortic morphological analysis.

In the practice of vascular surgery in our daily work, the expanded aortic morphology from two-dimensional (2D) plane provides practical information for surgical planning. The intrathoracic spatial shape of the aorta is streamlining like “?,” and the 2D image is generally regarded as the most instructive morphology for its superior performance in the unfolded state of the aorta (17, 18). Aortic view is defined as naked-eye recognition for a 2D plane image of the aorta (19). Clinicians can make operation plans referring to 2D images of the aortic view and predict the outcome of surgery based on the distorted shape of the blood vessels. In our previous research, the geometric projection method based on the aortic centerline was proposed for aortic morphology analysis (20–22). After 3D reconstruction and extraction of the centerline from the original CT image, the optimal single projection plane among the three orthogonal projection planes is manually selected based on the 2D primary plane projection (PPP) auxiliary diagnosis model for subsequent statistical analysis of morphological parameters of the aorta. However, the “optimal” projection plane obtained by subjective visual judgment is different from the natural shape of the aorta. In some cases, the spatial direction of blood vessels is too complex to use the method mentioned to analyze the aortic morphology.

Based on the researches above, this paper proposes a 3D rotation projection modeling method to solve the optimal projection plane. Furthermore, several morphological parameters have been induced to evaluate the projection plane and can provide an objective assessment method instead of subjective judgment. Then the optimal projection plane can be obtained by these parameters to characterize the actual shape of the aorta. This method contributes to the subsequent morphological analysis of aortic planar structure and formulation of AD operation planning and could provide a new idea in the study for the clinical treatment and prevention of AD.

## Materials and methods

### Population

This study is a retrospective study. The dissection group consists of patients diagnosed with Stanford A or B AD. Imaging data were retrieved from medical records of patients with continuous AD who underwent chest CT angiography (CTA) examination from January 2017 to December 2018. A Toshiba 64-slice CT scanner was used with a scanning range from the entrance of the thorax to the lower boundary of the lung. The scanning parameters were as follows: a tube voltage of 80–120 kV, a slice thickness of 0.5 mm, and a pitch of 1.0. The exclusion criteria for the dissection group were as follows: (1) patients with connective tissue disease (Marfan’s disease, Loeys-Dietz syndrome, or Ehlers-Danlos syndrome); (2) iatrogenic dissection; (3) patients with other aortic diseases such as aneurysms; (4) patients with previous aortic surgery; (5) patients with a previous cardiothoracic disease or cardiothoracic surgery; (6) patients with diseases that might distort the thoracic aortic morphology (pulmonary nodules with a diameter > 3 cm, mediastinal masses or lymph nodes with a diameter > 1 cm, pneumothorax, pulmonary bullae with a diameter > 3 cm, history of thoracic and mediastinal surgery, etc.); and (7) patients with diseases that might distort the shape of the thoracic wall (scoliosis, barrel chest, pectus carinatum, history of spinal surgery, etc.).

Imaging data of the healthy control group were retrospectively retrieved from medical records of patients with healthy aorta who underwent chest CTA examination continuously from April 2018 to December 2018. The exclusion criteria for the healthy control group were: (1) patients with existing or previous aortic diseases such as AD, aneurysms, and coarctation of the aorta, etc. (2) patients with a previous cardiothoracic disease or cardiothoracic surgery; (3) patients with diseases that might distort the thoracic aortic morphology (pulmonary nodules with a diameter > 3 cm, mediastinal masses or lymph nodes with a diameter > 1 cm, pneumothorax, pulmonary bullae with a diameter > 3 cm, history of thoracic and mediastinal surgery, etc.); and (4) patients with diseases that might distort the shape of the thoracic wall (scoliosis, barrel chest, pectus carinatum, history of spinal surgery, etc.).

### Extraction of aortic centerline

**FIGURE 1:**
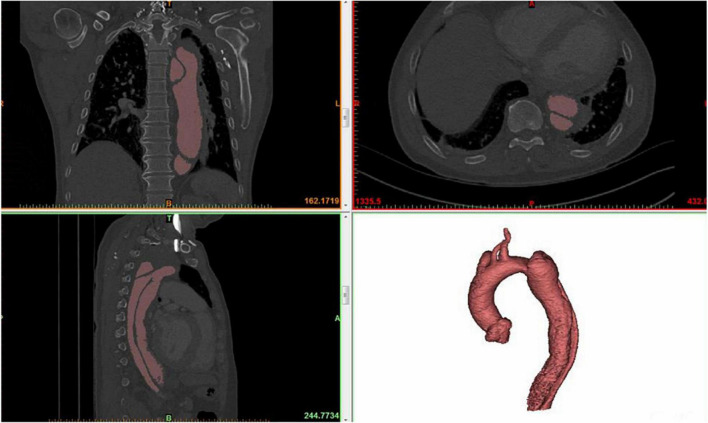
Medical image reconstruction of aortic morphology.

**FIGURE 2:**
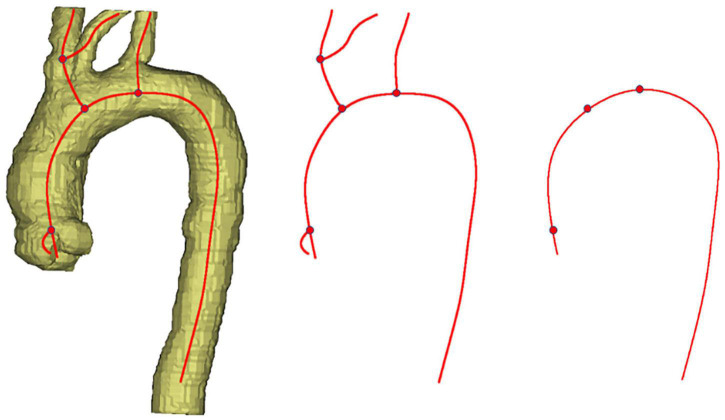
The extraction process of the aortic centerline.

All CT images were saved and output in the format of digital imaging and communication in medicine (DICOM). After being imported into the medical image reconstruction software, the images were converted into industry-standard files, which can realize the reconstruction of aortic 3D shape by gray threshold recognition. Figure 1 shows the original image information of CTA and the results after 3D reconstruction, from which the spatial morphological features of the aorta can be obtained obviously. Figure 2 presents the extraction process of the aortic centerline after 3D reconstruction. For the study of aortic morphology, the centerline is extracted from the reconstructed results to simplify the structural analysis and reserve the spatial characteristics of the aorta. Then the aorta is pruned, and the coordinates of the central line are output.

### Projection coordinate transformation of aortic centerline

In the traditional 2D PPP model for AD, the 3D information of the aorta only involves three primary projection planes. To improve the modeling method, the idea of 3D coordinate rotation is used to solve a more suitable 2D projection plane for subsequent morphological analysis.

**FIGURE 3:**
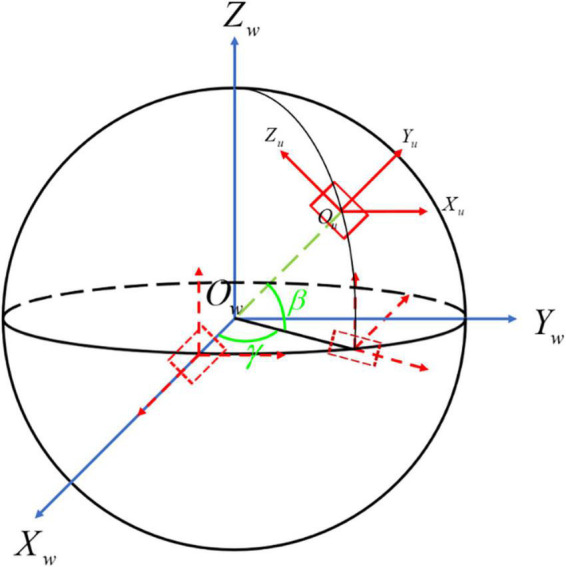
The transformation process of the aortic centerline. *O-X_w_Y_w_Z_w_* is the world coordinate system shown with the blue line, while *O-X_u_Y_u_Z_u_* is the user coordinate system presented with the red line. The dotted line denotes the process of 3D coordinate transformation from *O-X_w_Y_w_Z_w_* to *O-X_u_Y_u_Z_u_*.

The relationship between the two coordinate systems is shown in Figure 3. The world coordinate system O–X_*w*_*Y*_*w*_*Z*_*w*_ starts with the rotation around *Z_w_* by the angle of γ. Then the target coordinate system O–X_*u*_*Y*_*u*_*Z*_*u*_ can be obtained by the rotation around *Y_w_* with the angle of β. That is, the projection plane at any angle can be obtained *via* two-axis rotations of the world coordinate system, and the user coordinate system can be obtained through another axis rotation.

According to the selection of rotation axis *X_w_, Y_w_*, and *Z_w_*, the corresponding rotation angles are α, β, γ, respectively. The process of 3D coordinate rotation can be represented by a mathematical matrix. Generally, we define counterclockwise rotation around *X_w_*, clockwise rotation around *Y_w_* and counterclockwise rotation around *Z_w_* as the positive direction, individually (23). The matrices of rotation about three axes are as follows:

Rotate matrix around *X_w_*


(1) $$RotX=\left[\begin{array}{ccc} 1 & 0 & 0\\ 0 & cos\text{α} & -sin\text{α}\\ 0 & sin\text{α} & cos\text{α} \end{array}\right];$$

rotate matrix around *Y_w_*


(2) $$RotY=\left[\begin{array}{ccc} cos\text{β} & 0 & sin\text{β}\\ 0 & 1 & -sin\text{α}\\ -sin\text{β} & 0 & cos\text{β} \end{array}\right];$$

rotate matrix around *Z_w_*


(3) $$RotZ=\left[\begin{array}{ccc} cos\text{γ} & -sin\text{γ} & 0\\ sin\text{γ} & cos\text{γ} & 0\\ 0 & 0 & 1 \end{array}\right].$$

Suppose that the matrix *P* consists of a 3D point set *U*(*x, y, z*) with size of n × 3. The specific rotation process is as follows. First, transpose the matrix *P* to *P^T^*. Second, rotate the matrix *P^T^* around *X* axis. Third, let it rotate around *Y* axis. The final state of centerline can be obtained through the rotation of around *Z* axis with the left multiplication of *Z*, which is described as


(4) $$E=RotZ\cdot RotY\cdot RotX\cdot P^{T}.$$

It should be emphasized that the transformation from world coordinate system to user coordinate system must be carried out in strict accordance with the order above.

### Evaluation of the optimal projection plane

Due to the complexity of 3D morphological analysis of the aorta, the dimensionality can be reduced and projected to a specific plane. The plane is taken as the reference projection plane (RPP), on which the spatial winding of the aorta is the most minor, and the shape is the most unfolded. In view of the parameters for evaluation, contour length (CL), enclosed area (EA), and the sum of absolute residuals (SAR) are defined to quantitatively characterize the optimal projection plane and objectively evaluate the ability of 2D projection plane for the presentation of aortic morphology.

The CL is the length of the aortic 2D projection curve, which can be calculated approximately by summation of the distances between two points as follows:


(5) $$CL=\sum_{k=1}^{n}\sqrt{{\left(x_{k+1}-x_{k}\right)}^{2}+{\left(y_{k+1}-y_{k}\right)}^{2}}.$$

For the projection of the spatial curve to a specific projection surface parallel to the approximate plane composed of the aortic centerline, CL will be maximum on the optimal projection plane according to the Projection Theorem, defined as the maximum contour length (MCL) for the evaluation criterion.

**FIGURE 4:**
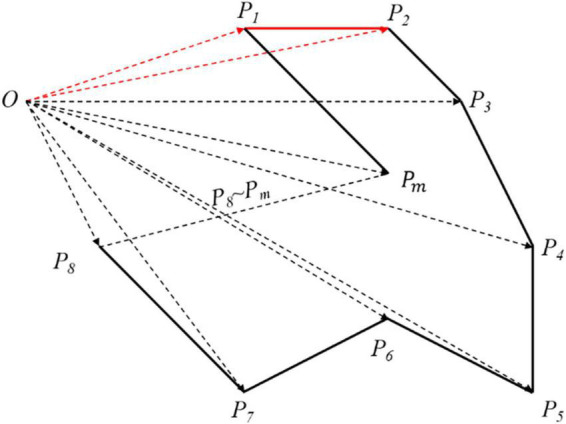
The area of arbitrary polygon. According to the origin *O*, the whole polygon has been divided into many vector triangles. Although the area solved is larger than what it is, every polygon has been processed in the same way so that it has no matters in the final result.

The EA is the 2D projected area of aorta. As shown in Figure 4, Ω is an m-polygon with vertex *P*_*k*_(*k* = 1, 2, 3, …, *m*, *m* ∈ N*), which is arranged in the positive direction along the boundary with the coordinates including *P*_1_ (*x*_1_, *y*_1_), *P*_2_ (*x*_2_, *y*_2_), …, *P*_*m*_ (*x*_*m*_, *y*_*m*_) in turn. The region vector graph of Ω is established, where a triangle consists of the origin *O* and any two adjacent vertices of Ω. For instance, the area of triangle △*OP*_1_*P*_2_ can be acquired by the outer product of two plane vectors $\vec{OP_{1}}$ and $\vec{OP_{2}}$ as follows:


(6) $$S_{\Delta OP_{1}P_{2}}=\frac{1}{2}\left(\vec{OP_{1}}\times \vec{OP_{2}}\right)=\frac{1}{2}(x_{1}y_{2}-x_{2}y_{1}).$$

Therefore, the area formula of arbitrary polygon is expressed as:


(7) $$EA=S_{\Omega }=\sum_{k=1}^{m-2}S_{\text{△}OP_{k}P_{k+1}}=\frac{1}{2}\sum_{k=1}^{m-2}\left(x_{k}y_{k+1}-x_{k+1}y_{k}\right).$$

In general, the first (last) vertex of the graph is regarded as the coordinate origin. Here, we select the first vertex of the graph as the origin *O*. When the optimal projection plane is obtained, the enclosing area of the projection curve reaches the peak, which is defined as the maximum enclosed area (MEA) for the evaluation criterion.

The residual is the deviation between the approximate plane formed by the rotated aortic centerline and RPP, and the coplanarity of those two above can be quantitatively characterized by SAR, which is expressed as


(8) $$SAR=\sum_{k=1}^{n}\left|x_{k}-\bar{x}\right|.$$

**FIGURE 5:**
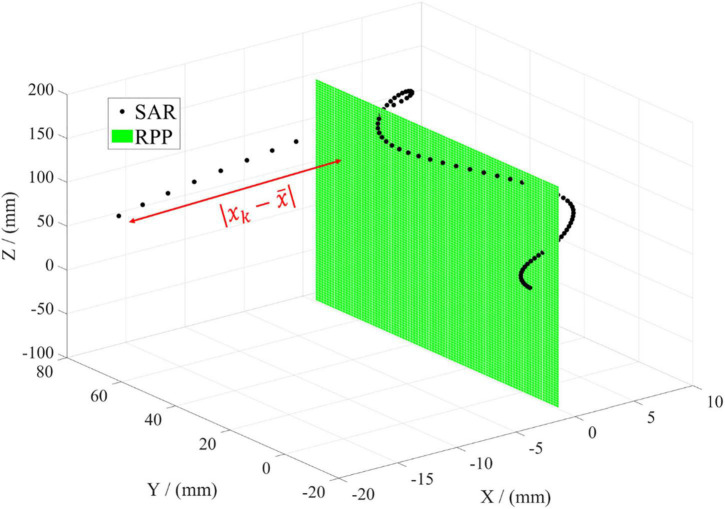
Illustration of evaluation parameter SAR. The black dot represents every data point of the aortic centerline in the spatial coordinate. The green plane denotes the reference projection plane (RPP). The red line means the distance between every data point and RPP.

For the projection on the *YOZ* plane as RPP, the deviation between the centerline and RPP can be calculated only considering the deviation in *X* direction. *X_k_* is the specific coordinate in *X* direction after rotation. $\bar{x}$ is the statistical mean of all data points, representing the approximate plane formed by the aortic centerline. Both of the two are shown in Figure 5. To simplify the subsequent analysis, the curve as a whole is translated so that $\bar{x}=0$. In this situation, *YOZ* plane is RPP, as shown in the green plane. The black points are the data coordinates of the aortic curve. The red line represents the distance between each data point and RPP (*YOZ*), which can characterize the dispersion degree between the two mentioned above. Each distance can be accumulated to figure out the parameter *SAR* as a characterization of the deviation of the aortic centerline from the projection plane. The smaller the deviation is, the better coplanarity of the 3D shape of the aorta, the smaller degree of spatial folding, and the better morphological expansion will be obtained.

As the spatial orientation of individual aortic centerline is complex, whether CL, EA, and SAR can be used as the evaluation parameters of the optimal projection plane needs further analysis and demonstration.

## Results

### Demographic characteristics

One hundred and forty samples were involved in the study, including 24 samples in type A AD (TAAD) group, 48 samples in type B AD (TBAD) group, and 71 samples in healthy control group. In the comparison of the three groups, the ratio of males in the healthy control group is lower [TAAD group: 87.5% (21/24); TBAD group: 84.4% (38/45); healthy control group: 52.1% (37/71), *P* < 0.001]. The age of patients in healthy control group is older (TAAD group: 54.13 ± 13.14 years; TBAD group: 57.64 ± 13.57 years; healthy control group: 63.99 ± 15.77 years, *P* = 0.007) The height of patients in TAAD group is higher (TAAD group: 173.38 ± 7.10 cm; TBAD group: 166.93 ± 7.63 cm; healthy control group: 163.10 ± 8.54 cm, *P* < 0.001). There is no significant difference in weight (TAAD group: 70.21 ± 16.65 kg; TBAD group: 66.96 ± 11.08 kg; healthy control group: 61.91 ± 12.45 kg, *P* = 0.12), hypertension rate [TAAD group: 75.0% (18/24); TBAD group: 71.1% (32/45); healthy control group: 57.7% (41/71), *P* = 0.179], diabetes rate [TAAD group: 8.3% (2/24); TBAD group: 0% (0/45); healthy control group: 7.0% (5/71), *P* = 0.169] and smoking rate [TAAD group: 4.2% (1/24); TBAD group: 11.1% (5/45); healthy control group: 19.7% (14/71), *P* = 0.129] in the three groups. Comparison of baseline data of groups was listed in Table 1.

**TABLE 1:** General characteristics of involved patients.

Characteristic	TAAD^a^ (24)	TBAD^b^ (45)	Healthy control (71)	*P*-value
Male	87.5% (21/24)	84.4% (38/45)	52.1% (37/71)	< 0.001
Age (year)	54.13 ± 13.14	57.64 ± 13.57	63.99 ± 15.77	0.007
Height (cm)	173.38 ± 7.10	166.93 ± 7.63	163.10 ± 8.54	< 0.001
Weight (kg)	70.21 ± 16.65	66.96 ± 11.08	61.91 ± 12.45	0.12
Hypertension	75.0% (18/24)	71.1% (32/45)	57.7% (41/71)	0.179
Diabetes	8.3% (2/24)	0% (0/45)	7.0% (5/71)	0.169
Smoke	4.2% (1/24)	11.1% (5/45)	19.7% (14/71)	0.129

^a^TAAD, type A aortic dissection.

^b^TBAD, type B aortic dissection.

### Quantitative evaluation of the optimal projection plane

In the traditional 2D PPP model for AD medical auxiliary diagnosis, clinicians usually select the specific plane closest to the actual situation from three primary projection planes (*XOY, XOZ*, and *YOZ*) as the optimal projection plane manually. In order to describe the optimal projection plane more objectively and accurately, three parameters, CL, EA, and SAR, are introduced as quantitative evaluation criteria. In the case of CL, it is the accumulation of the spatial distance of each data point on the aortic centerline. *CL*_3*D*_ characterizes the actual length of the aorta, and the length of the projection curve on three primary projection planes is represented by *CL*_*p*_. Therefore, the difference *D* between traditional PPP and the actual morphology of the aorta can be quantitatively characterized by


(9) $$D=CL_{3D}-CL_{p}.$$

When *D* is the minimum, CL of the centerline on the projection plane is close to the actual length of the aorta. This case is regarded as the optimal one. The other parameters, EA and SAR, are introduced to characterize traditional PPP, as shown in Table 2. The 3D length of the aortic centerline *CL*_3*D*_ = 294.79 mm by Equation (5). On *XOY* plane, *CL*_*p*_ gets the minimum with the value of 130.12 mm, and *D* reaches the maximum value of 164.67 mm by Equation (9), indicating a massive gap between the centerline on this projection plane and the actual situation. In this case, EA is the minimum and SAR is the maximum referring to Equations (7, 8). On *XOZ* plane, CL dramatically increases from 130.12 to 260.79 mm, and *D* greatly decreases from 164.67 to 34.10 mm. Meanwhile, the values of EA and SAR also follow the same trend. CL = 276.15 mm on *YOZ* plane (the maximum value), and *D* = 18.64 mm, which is the minimum value. It means that the curve projected on the *YOZ* plane is closest to the actual shape of the aortic centerline and has the optimal characterization. Therefore, the *YOZ* plane is the optimum under the traditional PPP model, which is consistent with the manual judgment of clinicians in artificial clinical diagnosis.

**TABLE 2:** Comparison between traditional 2D PPP and actual shape of aorta.

Groups	CL_*p*_/mm	D/mm	EA/mm^2^	SAR/mm
Actual^a^	294.79	0	/	/
XOY	130.12	164.67	114.04	3414.38
XOZ	260.69	34.10	2902.41	2659.84
YOZ	276.15	18.64	8258.38	1022.73

^a^The actual shape of aorta is 3D geometry instead of 2D planar structure, so it does not have EA and SAR.

The EA is positively correlated with the CL, and SAR is negatively correlated with CL and EA. The longer CL is, the larger EA will be for planar projection. This means the approximate plane formed by the centerline is more parallel to the projection plane, and the projection curve has better coplanarity. Thus, the curve has less spatial winding and more expanded geometry. It will obtain the optimal projection plane needed in the clinical diagnosis and treatment of AD. The results show that the three parameters CL, EA, and SAR can be used as evaluation criteria for the optimal projection plane in space.

### Single-axis rotation projection vs. primary plane projection

In the traditional PPP model, there is a distinct difference between the projection curve on a specific projection plane and the actual shape of the aortic centerline. This indicates that the primary projection plane as the optimal projection plane cannot fully characterize the actual aortic morphology. To reduce the difference, we rotate the centerline of the aorta around the axis by Equation (4) to solve a suitable projection plane in space as far as possible to be in line with the actual situation.

In the case of *YOZ* plane as RPP and CL of projection curve as the evaluation criterion, the MCL, EA, and SAR are compared with those of traditional PPP, as shown in Table 3. For rotation around the X-axis, MCL, EA, and SAR values are the same as those of PPP because the projection on *YOZ* plane (RPP) is not changed in the rotation process. For rotation around Y-axis, the values of three parameters are similar to those of PPP, demonstrating the negligible effect of this rotation. Compared to the former two methods, Z-axis rotation has better characterization. More specifically, its MCL is the largest, EA increases from 8258.38 to 8467.52 mm^2^, and SAR decreases from 1022.74 to 749.49 mm compared to traditional PPP. These results illustrate that the optimal projection plane, in this case, is more accurate than traditional PPP and has better performance in fitting with the actual aortic morphology.

**TABLE 3:** Comparison between single-axis rotation projection and traditional 2D PPP.

Groups	Angle α/rad	Angle β/rad	Angle γ/rad	MCL^a^/mm	EA/mm^2^	SAR/mm
Primary^b^	0	0	0	276.15	8258.38	1022. 74
X-axis	0.35	0	0	276.15	8258.38	1022. 74
Y-axis	0	3.09	0	276.39	8253.03	1074. 81
Z-axis	0	0	0.59	285.73	8467.52	749.49

^a^CL reaches its peak.

^b^Is the optimal plane of PPP.

### Three-dimensional rotation projection vs. single-axis rotation projection

Although the centerline projection by single-axis rotation is better than the traditional PPP, it is still different from the actual aortic geometry. By the idea of 3D coordinate transformation, the angle in the whole space can be searched by multi-axis rotation according to Equation (4). Consequently, a superior projection plane can be solved to characterize the actual geometry of the aorta further precisely compared to single-axis rotation. Table 4 shows the comparison between 3D rotation projection and single-axis rotation projection. As to 3D multi-axis rotation, there is little chance of MCL, but EA is larger, and SAR sharply decreases compared to single-axis rotation. Thus, the optimal projection plane of 3D rotation is superior to that of single-axis rotation.

**TABLE 4:** Comparison between 3D rotation projection and single-axis rotation projection.

Groups	Angle α/rad	Angle β/rad	Angle γ/rad	MCL/mm	EA/mm^2^	SAR/mm
Single-axis^a^	0	0	0.59	285.73	8467.52	749.49
3D^b^	0	3.12	2.55	285.73	8470.12	714.55

^a^The optimal plane of single-axis rotation is the one under Z-axis rotation.

^b^Is selected as the RPP of 3D rotation projection, which has no need of X-axis rotation.

### Optimal evaluation criterion for the optimal projection plane

**FIGURE 6:**
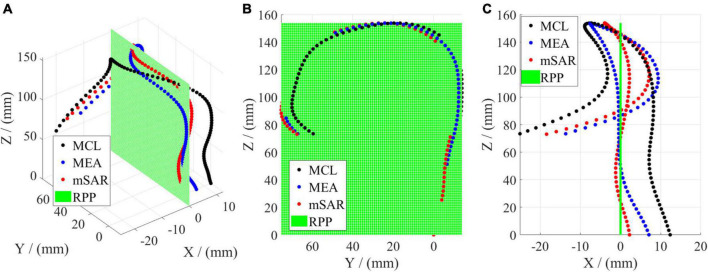
Comparison of three parameters of the optimal projection plane for 3D rotation. **(A)** Denotes the 3D geometry. **(B)** Shows the left elevation (*YOZ*). **(C)** Is the vertical view (*XOZ*).

The optimal projection plane evaluated by CL, EA, and SAR is shown in Figure 6, where the black curve denotes the state of maximum CL (MCL), the blue curve presents the state of maximum EA (MEA), the red curve shows the state of minimum SAR (mSAR). Figure 6A reveals the spatial shape corresponding to the above three parameters for evaluating the optimal projection plane. The projection morphology of the three is similar to the streamline of “?” as shown in Figure 6B, which meets the requirement of aortic profile morphology in the medical diagnosis of AD. The coplanarity between the three curves and the projection plane is shown intuitively in Figure 6C. The coplanarity between MCL and RPP is the worst, illuminating that morphological fluctuation is the strongest. Compared with the previous group, MEA has better coplanarity with RPP. This means the morphological fluctuation is gentler. According to Figure 6, the coplanarity of mSAR and RPP is optimal, which indicates that the undulation of the shape is the gentlest. So mSAR is the optimal choice as the evaluation criterion for the plane shape analysis of the aorta.

The quantitative characterization results of MCL, MEA, and mSAR are shown in Table 5. When MCL is taken as evaluating the projection, the corresponding EA value is less than that of MEA and mSAR. When MCL is taken as evaluating the projection, the corresponding EA value is less than that of MEA and mSAR. This indicates that the aortic centerline has a certain spatial entanglement. Although CL is the largest, the corresponding projection plane is not the optimal. When MEA is taken as the evaluation criterion, its rotation angle is quite different from that in *Y* direction of MCL. The difference between CL in the two cases is not apparent, but the SAR value of MEA is reduced to nearly half the value compared to that of MCL. Those results further show that the coplanarity of obtained spatial curves is not optimal in the case of MCL due to the orientation and winding of the centerline. For the projection method with mSAR as the evaluation criterion, both rotation angles differ from MCL and MEA. Compared with the former two methods, parameters EA and CL have little difference, but SAR is significantly lower than before. Figure 6C and Table 4 show that CL, EA, and SAR can characterize the optimal projection plane. Eventually, we choose SAR as the evaluation criterion of the optimal projection plane in space.

**TABLE 5:** Comparison of the evaluation parameters under 3D rotation projection.

Groups	Angle α/rad	Angle β/rad	Angle γ/rad	CL/mm	EA/mm^2^	SAR/mm
MCL	0	3.12	2.55	285.73	8470.12	714.55
MEA	0	3.12	2.81	283.69	8754.08	366.46
mSAR	0	3.05	2.74	284.21	8716.17	269.92

To verify the robustness of the proposed method, we chose the last zone of the aorta as an example according to the Society for Vascular Surgery (SVS) segmentation standards (24). Since Zone 5 is the mid descending aorta to the proximal edge of the celiac artery, the vascular segment presented nearly straight. Table 6 quantitatively shows the difference between traditional PPP and 3D rotation projection. The CL of the traditional PPP is 275.45, a little smaller than that of 3D rotation projection. Although the difference between traditional PPP and the proposed method has been greatly reduced, it still exists. However, there is an obvious difference in SAR of the proposed method and traditional PPP is 15.94 and 459.04 mm, respectively. As seen from Supplementary Video 2, the curve solved by the proposed method almost coincides with RPP, while that of traditional PPP has worse coplanarity with RPP. Thus, the 3D rotation projection method proposed in this manuscript has great robustness and universality, which may help obtain a stable, repeatable, and optimal projection shape of the aorta.

**TABLE 6:** Comparison between 3D rotation projection and 2D PPP in special situation^a^.

Groups^a^	CL/mm	EA^b^/mm^2^	SAR/mm
2D primary plane projection	275.45	/	459.04
3D rotation projection	283.64	/	15.94

^a^It is the case of Zone 5 of the aorta according to the SVS segmentation standards.

^b^Because of nearly straight shape, there is no closed pattern. Thus, there is no values of EA.

### Improvement of aorta tortuosity in three-dimensional rotation projection

In our previous research (22), the optimal projection plane can be determined among the three primary planes based on the traditional 2D PPP aided diagnosis model in medicine through subjective judgment. Then the centerline of the aorta was projected on this plane for statistical analysis of 2D morphology. In this paper, an improved projection algorithm based on 3D rotation is presented, including a reanalysis of the parameters—aortic tortuosity (AT) and its statistical validation. AT (22), characterizing the degree of aortic deformation can be used to distinguish normal people from patients with AD. Because of the morphological collapses, AT value of patients with AD is larger than normal.

One hundred and forty cases of clinical CT data were analyzed *via* the Wilcoxon signed-rank test mentioned in our article (22), which was used to validate the robustness of the two groups of samples, as shown in Table 7. Compared to normal people, AT value of AD patients is higher, and the degree of deformation is greater. These phenomena accord with the diagnosis of clinical medicine. Upon traditional PPP model, *P* = 0.015 (< 0.05). This result shows statistical significance between the two samples and good algorithm robustness and verifies the valuable reference of the parameter AT. Compared to the reported 2D PPP, the projection results of the 3D rotation projection method have the characteristics of longer CL, larger projection area, smaller space winding degree, and better geometric morphology. In particular, parameter AT with a bigger value illustrates a greater difference between patients with AD and those with normal. Meanwhile, *P* = 0.008 (<0.015), which means greater robustness, higher discrimination and a better representation effect.

**TABLE 7:** Robustness test results of aortic tortuosity.

	AD patients	Normal people	*P*-value
2D primary plane projection	3.10 ± 0.59	2.88 ± 0.53	0.015
3D rotation projection	3.16 ± 0.61	2.92 ± 0.53	0.008

For the same 140 cases of data, the Kolmogorov-Smirnov (K-S) test of two samples mentioned by Qiu (22) was used to test whether the morphological parameter AT can distinguish AD patients from normal people (as shown in Table 8). According to the traditional 2D PPP method, the approximate bilateral value of *P* is 0.106 (>0.05), while that of the proposed 3D rotation projection method is 0.063 (>0.05). Although our method increases the difference, there is still no significant difference in statistics. To further confirm the efficiency of the parameter AT and the K-S analysis, we used another common nonparametric test, named Mann-Whitney *U* test. The bilateral P value of our proposed method is 0.009 (<0.01), as half as that of the traditional method. The results above illustrate that aortic tortuosity (AT) can be used as a new morphological parameter to distinguish patients, and moreover our method will improve its differentiation between AD patients from the normal.

**TABLE 8:** Two sample non-parametric test for aortic tortuosity.

	*K-S* test		Mann-Whitney *U*-test	
	*Z*-value	*P*-value	*Z*-value	*P*-value
2D primary plane projection	1.211	0.106	−2.361	0.018
3D rotation projection	1.316	0.063	−2.620	0.009

## Discussion

Endovascular therapy has developed rapidly in recent years. As one of the most difficult challenges in vascular diseases, AD is one of the crucial concerns of endovascular treatment research. AD image monitoring in endovascular treatment includes the following four parts: (1) Monitoring of aortic morphology before the onset of AD, exploring morphological risk factors of AD. (2) Before the endovascular therapy, the position of stent placement was designed according to the morphology of the aorta. (3) During endovascular treatment, the procedure can be adjusted continuously based on the morphology of the aorta shown on the digital subtraction angiography (DSA). (4) After surgery, it is necessary to monitor the morphology of the aorta and observe the deformation of the aortic shape to predict the long-term prognosis. Although 3D aortic morphology and raw CTA profile contain richer information (25, 26), an intuitive and clinically meaningful parameter is necessary for rapid diagnosis and preoperative decision-making. In the imaging monitoring scenes mentioned above, 3D information can be replaced by the 2D shape of the aorta in most cases, which avoids complex 3D morphological parameters and expresses intuitively. However, finding the appropriate 2D aortic plane that can best reflect the information among the 3D aortic morphology is a challenge rarely discussed before. Therefore, we are committed to obtaining a better quality 2D projection plane to describe the morphology of the aorta more accurately and understandably.

As the aorta is a pipeline, most clinicians use the aortic centerline as the target of clinical intervention (17, 27–29). The centerline of the aorta can preserve the geometric morphological characteristics of the aorta and minimize the influence of other factors such as vascular branches and aortic diameter by using geometric mathematical methods (30). Additionally, since centerline measurement is helpful for establishing a surgical protocol in clinical practice, current centerline extraction methods are readily available and reliable (19, 31, 32). The centerline method is also used in our study, which effectively removes the morphological factors such as dissecting false lumen and branch arteries and highlights the elongation and distortion of the aorta. This algorithm is supposed to be intuitive and reflects the pathological features of the dissected aorta, which helps differentiate the dissected aorta from the healthy aorta (33, 34). Thus, it provides a potential scheme for the follow-up morphological analysis of the aorta. The centerline extraction is used in our study and effectively reduces the interference factors such as dissecting false lumen and branch arteries and highlights the elongation and distortion of the aorta. It provides a potential scheme for the follow-up morphological analysis of the aorta.

**FIGURE 7:**
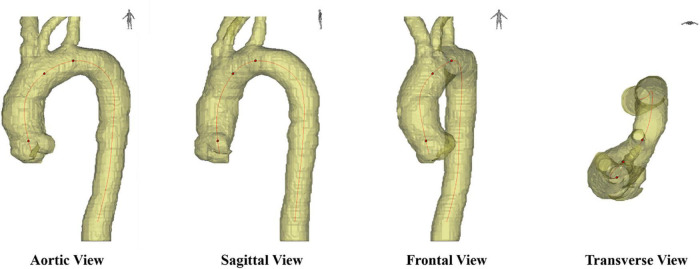
Different axial planes in medical images, including sagittal view, frontal view, transverse view and aortic view.

The critical issue is how to obtain a 2D projection shape that can represent the actual geometry of the aortic centerline. The shape of the aorta is different when viewed from different directions, as shown in Figure 7. when looking at the aorta from the aortic view (the left-anterior oblique 45° for most patients), it has the most prominent unfolding shape and is the easiest to observe a change in morphology. In general, clinicians judge aortic view manually, and the subjective error is too large, and it takes time and effort. On top of that, the shape of the aorta of patients will change over time, especially after surgical treatment, and the stent will cause deformation in the blood vessel. In the absence of a standard expanded plane, it is hard to compare preoperative and postoperative CTA images of the patients, or to compare CTA images among different patients. Therefore, the issue we want to solve is how to stably, quantitatively obtain the optimal 2D plane image of the aortic centerline as the new aortic view.

Converting the 3D shape of aorta to a 2D shape is a data dimensionality reduction problem. As for the proposal of different optimization parameters, CL has been a classic parameter used to describe aortic morphology. Along with other parameters such as aortic arch symmetry and angulation, length is thought to identify the principal model of variation in aorta (18), represent aortic aging (35), and AD (36). According to the definition of the existing criterion, CL, from geometry and modeling, the larger CL is, the less spatial winding the aortic centerline will have, and the closer it is to the solution of the optimization problem above. In this manuscript, we define new parameters including EA and SAR with similar properties. In order to obtain the optimal solution, we verify and compare the three parameters, CL, EA, and SAR. The results show that the approximate solution to the optimization problem can be obtained using the three parameters, and that using SAR as the evaluation has the optimal solution.

In this paper, we propose a novel method to quantitatively characterize the aortic view, which can stably and repeatably obtain the aortic view of each case through the algorithm. Our method can provide a measurable plane at any phase to compare the changes in the shape of the aorta and complete the condition assessment for the patient. Furthermore, compared to the PPP model, the deformation and distortion of the aorta are presented more intuitively, and it only takes about 1 s (as seen in Supplementary Videos 1–3), which is efficient and straightforward. Even in more extreme situations, such as a particular case of the Kommerell Diverticulum, the optimal shape of aortic 2D projection will be obtained using the proposed method, as shown in Supplementary Video 3. Because of the congenital malformation, there was a large distortion in the shape of aorta. In the traditional PPP model, under the view of left-anterior oblique 45°, it seemed to have normal morphology. However, as it had complicated spatial distortion, there was obvious rigidity between Zone 1 and 2 (from the distal edge of the innominate to the distal edge of the left subclavian) under the aortic view solved by our method. The shape obtained and its reasons behind may be further explored in the later work.

It is noteworthy that in such studies on the prevention of morphological lesions of the aorta, compared with the CT image quality, promotion should be the primary consideration since it requires more datasets of clinical cases. The higher the CT image quality is, the greater the dose of radiation used and the greater the damage to the human body. This is not allowed in clinical ethics. Furthermore, obtaining a high-quality reconstruction image needs more costs, including expert reconstruction and software copyright, which does no good to the practical promotion in hospital departments. In conclusion, a low-quality image means lower cost and better promotion, whether it is the original CT image or the reconstructed image.

Although an application scenario of this method has been demonstrated through the above work, it still has some limitations. Since the base is relatively small, the clinical data of 140 cases we collected were not classified according to physiological indicators. Retrospective and even prospective collection of more AD patients and healthy human aortic CTA data is needed. As the sample size increases, it will be possible to improve our algorithm. In addition, considering that the AD CTA data were derived from patients already with AD while the control CTA data were from another healthy population, it may not further explore the specific effects of AD on aortic morphology. Prospective collection of CTA data before and after the AD onset will provide more information and compensate for this deficiency. Finally, we believe that this method is suitable as a pretreatment method, and new findings and conclusions may be drawn in combination with other aortic 2D morphology studies. For this method to be better applied to practice, future researchers are supposed to investigate more efficient algorithms and concise methods. With the deepening of the integration of medical and engineering disciplines, cardiovascular therapy will be supposed to provide various parameters of the aorta, including diameter, length, curvature, radial lines of different cardiac cycles, and even mechanical characteristics based on hardware, algorithm, artificial intelligence, and deep learning, and the results should be reproducible.

## Conclusion

In this paper, we presented a planar geometric projection modeling method based on 3D coordinate rotation. The coordinates of the aortic centerline were obtained by 3D reconstruction of aortic images from chest CT. The CL, EA, and the SAR have been defined to evaluate the result of traditional PPP quantitatively. These methods are superior to artificial subjective judgment. In addition, the traditional PPP model has been improved by uniaxial rotary projection and 3D rotary projection algorithms proposed in this work. Consequently, the centerline projection obtained by the 3D multi-axis rotation method using SAR as the evaluation criterion had the best representation of the actual morphology of the aorta. The effectiveness and applicability of our method in the analysis of AD geometry were verified by statistical analysis of AT of the same batch of data.

The method proposed in this paper can work as a pretreatment of other morphological analyses of AD with simplicity and visuality, which may have an expectation for the applicability of specific analysis of AD and other aortic diseases. This method will be a reliable, comprehensive, big-data research mode, which may provide a technical method for the prevention and diagnosis of AD in the future.

## Data availability statement

The original contributions presented in this study are included in the article/Supplementary material, further inquiries can be directed to the corresponding author.

## Ethics statement

The studies involving human participants were reviewed and approved by the Medical Ethics Committee of Shanghai Ninth People’s Hospital Affiliated to Shanghai Jiao Tong University School of Medicine and Anhui Medical University. Written informed consent for participation was not required for this study in accordance with the national legislation and the institutional requirements.

## Author contributions

TP, XL, and JHZ: conceptualization. TP, HP, PQ, HY, ZJ, HM, and JLZ: methodology. TP: software. HP and PQ: validation. TP, HP, and PQ: formal analysis and writing—original draft preparation. TP, KC, YZ, RS, YW, BZ, and YY: investigation. XL, PQ, SF, and JHZ: resources and supervision. All authors have read and agreed to the published version of the manuscript.
